# Supplementary material for: Actinide inverse trans influence versus cooperative pushing from below and multi-center bonding
Source: Nat Commun. 2023 Jul 18;14:4307. doi: 10.1038/s41467-023-39626-8 (PMC10354014; doi:10.1038/s41467-023-39626-8)
Supplement: Supplementary file 1 — Supplementary Information [file 41467_2023_39626_MOESM1_ESM.pdf]

Supplementary Information for Actinide Inverse Trans Influence versus Cooperative Pushing from Below and Multi-Center Bonding

Laura C. Motta[1,2], Jochen Autschbach[1*]

[1]Department of Chemistry
University at Buffalo
State University of New York
Buffalo, NY 14260-3000, USA
email: jochena@buffalo.edu

[2]Present affiliation:
Department of Marine Chemistry & Geochemistry
Woods Hole Oceanographic Institution
Woods Hole, MA 02543-1050, USA.

June 21, 2023

List of Supplementary Tables

1 NRT weights of the Lewis structures 1 and 2, and the associated non-Lewis RMSD errors in the NRT compared to using only a single resonance structure 1 or 2. DFT PBE, PBE0, and TPSS calculations. 3
2 Orbital interaction energies ΔE_{orb} for the dominant bonding interactions. DFT PBE, PBE0, and TPSS calculations. 4
3 Calculated (DFT/B3LYP) An–terminal bond orders. 5
4 NRT weights of the CUO Lewis structures 1 and 2^a, and the associated non-Lewis RMSD errors in the NRT compared to using only a single resonance structure 1 or 2, at the DFT PBE0 and B3LYP geometries. 6
5 Orbital interaction energies ΔE_{orb} for the dominant bonding CUO interactions at the DFT PBE0 and B3LYP geometries 7

List of Supplementary Figures

1 Occupied orbitals are red and blue and virtual orbitals are orange and yellow. MPS natural occupation numbers are given in purple for O−U and in red for C−U entangled orbitals. The area of the red circles is proportional to an orbital's single-orbital entropy, while the thickness of connecting lines is proportional to the mutual orbital pair information. Orbital isosurface values are ±0.04. ^{a}PBE0 C−U(Å) = 1.733 and U−O = 1.779. ^{b}B3LYP C−U(Å) = 1.746 and U−O = 1.801. 8

Supplementary Tables 1 to 5

Supplementary Table 1: NRT weights of the Lewis structures 1 and 2, and the associated non-Lewis RMSD errors in the NRT compared to using only a single resonance structure 1 or 2. DFT PBE, PBE0, and TPSS calculations.

	**Weights**^{a}		**RMSD**^{b}		
**Compound**	**1**	**2**	only **1**	only **2**	**NRT**
$[UOU]^{2+c}$ PBE	91	-	0.37	–	–
$[UOU]^{2+c}$ PBE0	98	-	0.35	–	–
$[UOU]^{2+c}$ TPSSh	99	-	0.35	–	–
$(R^a)_3NU^{VI}N$ PBE	46	54	1.96	1.95	0.10
$(R^a)_3NU^{VI}N$ PBE0	46	54	1.84	1.83	0.09
$(R^a)_3NU^{VI}N$ TPSSh	45	55	1.87	1.86	0.09

^{a}Percent weights of Lewis structures 1 and 2 of Fig. 1C and D in the resonance stabilized electronic structure according to NRT. ^{b}Non-Lewis RMSD (number of electrons). ^{c} The dominant structure for uranyl is $O{\equiv}U{\equiv}O^{+2}$.

Supplementary Table 2: Orbital interaction energies ΔE_{orb} for the dominant bonding interactions. DFT PBE, PBE0, and TPSS calculations.

**Compound**	ΔE_{orb} σ^2	π	σ^1
$[\text{OU}^{\text{VI}}{-}\text{O}]^{2+}$ PBE	-321.4	-176.6	-15.7
$[\text{OU}^{\text{VI}}{-}\text{O}]^{2+}$ PBE0	-324.1	-170.1	-16.0
$[\text{OU}^{\text{VI}}{-}\text{O}]^{2+}$ TPSSh	-322.9	-175.0	-14.0
$(\text{R}^{\text{a}})_3\text{NU}^{\text{VI}}{-}\text{N}$ PBE	-276.7	-163.0	-25.5
$(\text{R}^{\text{a}})_3\text{NU}^{\text{VI}}{-}\text{N}$ PBE0	-292.5	-153.0	-27.5
$(\text{R}^{\text{a}})_3\text{NU}^{\text{VI}}{-}\text{N}$ TPSSh	-288.2	-159.4	-26.7

The ΔE_{orb} for the two π-bonding interactions are equivalent and only one is shown.

Supplementary Table 3: Calculated (DFT/B3LYP) An–terminal bond orders.

**Compound**	**Mayer**	**Nalewajski-Mrozek**	**Covalent Gopinathan-Jug**
$[U^{V}{-}N]^{2+}$	3.1	4.5	3.3
$OTh^{IV}{-}O$ C_{2v}	1.9	2.7	2.0
$OTh^{IV}{-}O$ $D_{\infty h}$	2.0	2.6	1.9
$[OU^{VI}{-}O]^{2+}$ C_{2v}	2.4	3.2	2.8
$[OU^{VI}{-}O]^{2+}$ $D_{\infty h}$	2.3	3.2	2.7
$[ONp^{VII}{-}O]^{3+}$	2.4	3.2	2.7
$[OU^{V}{-}O]^{1+}$	2.2	3.1	2.5
$[ONp^{VI}{-}O]^{2+}$	2.3	3.2	2.7
$NU^{VI}{-}N$	3.1	4.0	3.2
$OU^{VI}{-}C$	3.5	4.4	3.5
$(R^{a})_3NTh^{IV}{-}Cl$	0.9	1.2	0.8
$[(R^{a})_3NTh^{IV}{-}N]^{2-}$	2.9	3.4	2.7
$(R^{a})_3NU^{VI}{-}N$	3.0	3.6	3.2
$[(R^{a})_3NU^{V}{-}N]^{1-}$	3.0	3.9	3.1
$[(R^{a})_3NU^{IV}{-}N]^{2-}$	3.0	4.1	3.0
$(R^{a})_3NU^{V}{-}O$	2.0	2.9	2.4
$(R^{a})_3NNp^{V}{-}O$	2.0	3.1	2.4
$MeU^{VI}(R^{b})_3{-}O$	1.9	2.7	2.4
$PhCCU^{VI}(R^{b})_3{-}O$	1.9	2.7	2.4
$CU^{VI}{-}O$*	2.1	3.3	2.4
$NU^{VI}(R^{a})_3{-}N$*	0.4	0.5	0.4
$O(R^{b})_3U^{VI}{-}Me$*	0.7	1.1	0.9

*BO analysis of the *trans* An—Ligand bond.

Supplementary Table 4: NRT weights of the CUO Lewis structures 1 and 2[a], and the associated non-Lewis RMSD errors in the NRT compared to using only a single resonance structure 1 or 2, at the DFT PBE0 and B3LYP geometries.

	**Weights**[a]		**RMSD**[b]		
**Compound**	**1**	**2**	only **1**	only **2**	**NRT**
CUO[c] PBE0	72	28	0.29	0.45	0.07
CUO[d] B3LYP	75	26	0.28	0.44	0.07

[a]The resonance structure for CUO is [(1) C≡U≡O: ⟷ (2) :C≡U≡O]. [b]Non-Lewis RMSD (number of electrons). [c]PBE0 C−U(Å) = 1.733 and U−O = 1.779. [d]B3LYP C−U(Å) = 1.746 and U−O = 1.801

Supplementary Table 5: Orbital interaction energies ΔE_{orb} for the dominant bonding CUO interactions at the DFT PBE0 and B3LYP geometries

		ΔE_{orb}	
**Compound**	σ^2	π	σ^1
CUO[a] PBE0	-487.1	-321.3	-23.7
CUO[b] B3LYP	473.0	-317.2	-24.6

The ΔE_{orb} for the two π-bonding interactions are equivalent and only one is shown. [a]PBE0 C—U(Å) = 1.733 and U—O = 1.779. [b]B3LYP C—U(Å) = 1.746 and U—O = 1.801

Supplementary Figure 1

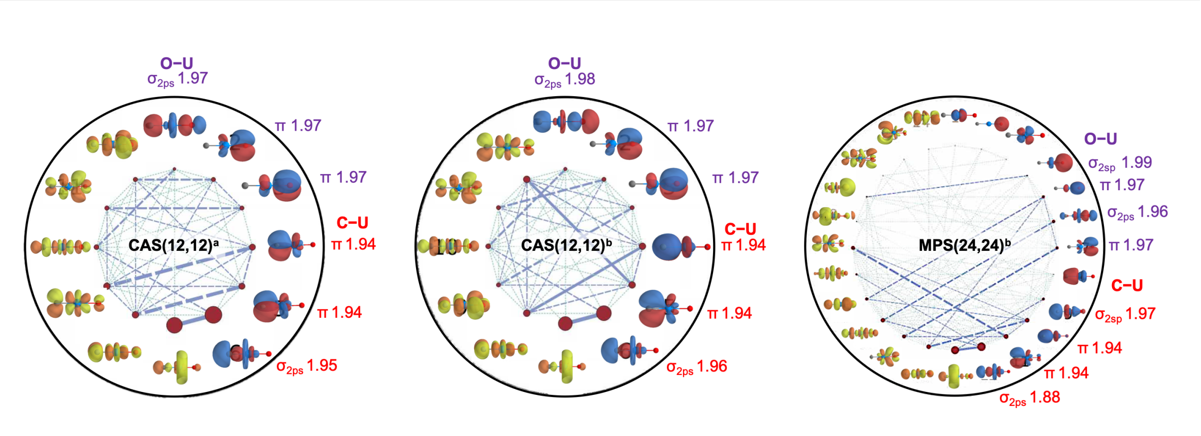

Supplementary Figure 1: Occupied orbitals are red and blue and virtual orbitals are orange and yellow. MPS natural occupation numbers are given in purple for O−U and in red for C−U entangled orbitals. The area of the red circles is proportional to an orbital's single-orbital entropy, while the thickness of connecting lines is proportional to the mutual orbital pair information. Orbital isosurface values are ±0.04. [a]PBE0 C−U(Å) = 1.733 and U−O = 1.779. [b]B3LYP C−U(Å) = 1.746 and U−O = 1.801.
